# Altering Transplantation Time to Avoid Periods of High Temperature Can Efficiently Reduce Bacterial Wilt Disease Incidence with Tomato

**DOI:** 10.1371/journal.pone.0139313

**Published:** 2015-10-06

**Authors:** Zhong Wei, Jian-Feng Huang, Jie Hu, Yi-An Gu, Chun-Lan Yang, Xin-Lan Mei, Qi-Rong Shen, Yang-Chun Xu, Ville-Petri Friman

**Affiliations:** 1 Jiangsu Provincial Key Lab for Organic Solid Waste Utilization, National Engineering Research Center for Organic-based Fertilizers, Nanjing Agricultural University, Weigang 1, Nanjing, PR China; 2 Institute of Agricultural Resources and the Environment, Guangdong Academy of Agricultural Sciences/Guangdong Key Laboratory of Nutrient Cycling and Farmland Conservation, Guangzhou, PR China; 3 University of York, Department of Biology, Wentworth Way, York, YO10 5DD, London, United Kingdom; Virginia Tech, UNITED STATES

## Abstract

Tomato bacterial wilt caused by *Ralstonia solanacearum* bacterium is a severe problem in Southern China, where relatively high environmental temperatures commonly prevails during the crop seasons. Previous research has indicated that bacterial wilt disease incidence generally increases during the warm months of summer leading to reduced tomato yield. Moreover, the efficacy of bio-organic fertilizers (BOFs)–organic compost fortified with pathogen-suppressive bacteria—is often lost during the periods of high environmental temperatures. Here we studied if the disease incidence could be reduced and the BOF performance enhanced by simply preponing and postponing the traditional seedling transplantation times to avoid tomato plant development during periods of high environmental temperature. To this end, a continuous, two-year field experiment was conducted to evaluate the performance of BOF in two traditional (late-spring [LS] and early-autumn [EA]) and two alternative (early-spring [ES] and late-autumn [LA]) crop seasons. We found that changing the transplantation times reduced the mean disease incidence from 33.9% (LS) and 54.7% (EA) to 11.1% (ES) and 7.1% (LA), respectively. Reduction in disease incidence correlated with the reduction in *R*. *Solanacearum* pathogen density in the tomato plant rhizosphere and stem base. Applying BOF during alternative transplantation treatments improved biocontrol efficiency from 43.4% (LS) and 3.1% (EA) to 67.4% (ES) and 64.8% (LA). On average, the mean maximum air temperatures were positively correlated with the disease incidence, and negatively correlated with the BOF biocontrol efficacy over the crop seasons. Crucially, even though preponing the transplantation time reduced the tomato yield in general, it was still economically more profitable compared to LS season due to reduced crop losses and relatively higher market prices. Preponing and postponing traditional tomato transplantation times to cooler periods could thus offer simple but effective way to control *R*. *solanacearum* disease outbreaks.

## Introduction

Bacterial wilt is one of the most devastating diseases of tomato in tropical and subtropical regions of the world. It is caused by the *Ralstonia solanacearum* (Smith) species complex, which is a soil-borne pathogen notorious for its virulence, broad host range, and wide geographic distribution [1]. Moreover, once successfully invaded to tomato fields, eradication of *R*. *solanacearum* is very difficult, because it can survive in soil, water or reservoir plants for long periods before coming into contact with a new host [2]. The disease process is very complex and involves several stages, which are dependent on environmental conditions, the physiological state of the pathogen, and the host. Ultimately, pathogen invades host vascular tissues, where it multiplies until it blocks the xylem and causes wilt symptoms. To control the disease, integrated control strategies that prevent pathogen invasion in the roots and the above-ground parts of host plants has been suggested [3–5]. These strategies include liming, fertilization, crop rotation and chemical controls [6,7]. Unfortunately, these strategies are often unable to reduce crop losses down to tolerable levels. Moreover, although chemical soil disinfection can temporarily eradicate most microbial flora, pesticide-resistant pathogens may rebound leading to ever more problematic disease outbreaks [7,8].

Environmentally friendly alternatives for pesticides, such as bio-organic fertilizers (BOFs), have been developed to better control soil-borne diseases [9]. BOFs are prime examples of next-generation sustainable agriculture. They are manufactured from agricultural waste products and thus reduce environmental pollution. Application of BOF could reduce the amount of inorganic fertilizer needed in crop production. Furthermore, BOFs can be fortified with some specific antagonistic microorganisms that suppress target pathogens in the soil [10]. Thus far, BOFs have been reported to be efficient in controlling soil-borne diseases, such as Fusarium wilt, damping off, bacterial wilt, and black shank [11,12]. However, the biocontrol effects of BOFs on tomato bacterial wilt have been inconsistent in field trials and it is thought that the environmental temperature could affect the efficacy of disease suppression by BOFs [13–16].

Environmental temperature could drive bacterial wilt outbreaks by directly affecting the pathogen invasion success [16]. For example, elevated temperatures have been found to increase the severity of bacterial wilt in *R*. *solanacearum*-resistant tomato plants in environmental chambers [16]. Moreover, pathogen density and disease incidence have been shown to correlate positively in two-year long field experiments with tomato [17,18]. However, even though some *R*. *solanacearum* biovars are specialized to cause bacterial wilt in high environmental temperatures [16], lots of inter-strain variation exists [19]. For example, Race 3, Biovar 2 (R3B2) strains can cause bacterial wilt in potatoes at temperatures as low as 16°C [20]. Besides having effect on pathogen, elevated temperatures could also affect the biocontrol efficacy of bio-organic fertilizers. Bacterial species belonging to genus *Bacillus* are commonly used as biocontrol agents because they are able to inhibit wide array of pathogenic microorganisms via production of antibacterial and antifungal compounds [21,22]. The production of antibiotic molecules by *Bacillus* bacteria depends on the environmental temperature, which could thus affect the efficacy of BOFs [16]. Furthermore, plant immune response to pathogens can differ depending on the environmental temperature [23,24], which could also affect the pathogen invasion success.

Here we focused on testing a simple ecological approach to reduce crop losses: preponing and postponing traditional transplantation times to avoid tomato plant development during periods of high environmental temperatures when the risk of bacterial wilt outbreak is highest and when the BOF biocontrol efficacy is the lowest [25]. Typical spring and autumn crop seasons in Nanjing last from February to June and from July to November, respectively, and tomato crop losses are especially high during relatively warmer autumn season [16]. We hypothesized that earlier and later transplantation times should result in lower bacterial wilt incidence if the pathogen growth in the soil and tomato stem is suppressed by lower temperature during crop season. Moreover, lower mean temperatures could potentially increase the efficiency of BOF biocontrol [16] and decrease the bacterial wilt disease incidence even further. To study this hypothesis, we set up a two-year long field experiment where we manipulated the transplantation time of both spring and autumn seasons. Specifically, we preposed the transplantation time of spring season with ~2–2.5 months and postponed the transplantation time of autumn season with ~1–1.5 months and compared these treatments with traditional spring and autumn transplantation times. To study how BOF biocontrol efficacy depended on the transplantation time, half of the plants were treated with BOF at seedling phase and other half were left untreated. Pathogen infection was let to occur naturally as the field has previously been shown to be contaminated by *R*. *solanacearum* race 1 biovar 3 on the basis of pathogen host range and carbon utilization patterns [16]. Bacterial wilt incidences were recorded in the end of all crop seasons, *R*. *solanacearum* pathogen densities measured both in the rhizosphere soil and in the tomato plant stems and average tomato yield and farmer income estimated for each year per every treatment.

## Materials and Methods

### Preparation of bio-organic fertilizer (BOF)

A BOF was prepared as described by Wei et al. [16], with some modifications. In the previous study, we used both rapeseed cake compost (RCC) and pig manure compost as a carrier mixture [16]. Further research has shown that pig manure compost facilitates *R*. *solanacearum* growth, whereas RCC inhibits it. As a result, only rapeseed cake compost was used in this study. The compost was fortified with *Bacillus amyloliquefaciens* QL–18 strain, which has previously been shown to be antagonist against *R*. *solanacearum* [16]. The *B*. *amyloliquefaciens* QL–18 strain was provided by the Jiangsu Provincial Key Lab of Organic Solid Waste Utilization and it was routinely cultured on LB medium plates [16]. The final concentration of QL–18 in the fortified RCC was approximately 10^9^ cells (CFU) g^−1^ and this final mixture was designated as BOF.

### Field experiment

The field experiment study site (no other specific permissions were required for these locations, and the field studies did not involve endangered or protected species) is located in the town of Qilin (118° 57' E, 32° 03' N; previously described in detail in Wei et al. [16]). Qilin is an important vegetable production base for nearby urban population of Nanjing, China. Recently, bacterial wilt of tomato has become a widespread problem in Qilin. Two tomato (*Solanum lycopersicum* Mill) crops can be grown per year at this location. For this study, we chose early- and late-spring crop seasons (ES and LS) and early- and late-autumn crop seasons (EA and LA), as shown in S1 Table. The tomato seedlings (cultivar Hezuo 903) were first grown in nursery trays for 30 (autumn) or 45 (spring) days before transplantation to the field. Tomato seedlings were grown with a nursery substrate (commercially available from Huaian Agricultural Technological Development Ltd., Huanyin, Jiangsu, China). Half of the seedlings received 1% (w/w) of BOF, which was mixed with the nursery substrate before sowing. The other half of the seedlings did not receive BOF and were assigned to the control treatment. BOF was not reapplied when the plants were transferred to the field.

Field trials were conducted during two consecutive years from January 2011 to December 2012. No pesticides were used and standard chemical fertilization was applied. Four different transplantation treatments were used (ES, LS, EA and LA) and every transplantation treatment was further divided into BOF and no-BOF (control) treatments for each year (total of 8 treatments per year). The trial was conducted following a randomized block design within every crop season treatment. Five independent replicate plots (blocks) were used for both no-BOF and BOF treatments in random block design and 108 plants were planted per each plot (1.8×9 m area). Within each plot, plants were spaced evenly at approximately 30 cm apart into 4 rows each containing 27 plants. Pathogen infection was let to occur naturally as the field has previously been shown to be contaminated by *R*. *solanacearum* [16]. Disease development was expressed as the disease incidence (DI), which denotes the percentage of wilted plants on the first day of the harvest (S1 Table). The decision to focus only on one time point was made based on our previous experience on *Ralstonia* wilt disease development: most damage will take place before the first date of harvest and subsequent wilting has only minor effect on tomato yield. The biocontrol efficacy (BCE) of treatment with BOF was calculated for each crop season using the following equation: BCE = (DI of control treatment—DI of BOF treatment) / DI of control treatment × 100%.

### Measuring *R*. *solanacearum* densities in tomato plant rhizosphere and stem base

To determine *R*. *solanacearum* densities, rhizosphere soils were collected from three randomly selected tomato plants per plot on the first day of harvest. Excess soil was gently shaken from the roots, and the remaining soil that adhered closely to the roots was considered as rhizosphere soil. Five grams of rhizosphere soil was added to 45 mL of sterilized water and shaken for 30 min on a rotary shaker. Several 10-fold dilutions were made, and 0.1-mL aliquots were spread on the surface of modified semi-selective medium (M-SMSA) [16]. After 3 days of incubation at 30°C, bacterial densities were measured as the number of colonies per gram of soil (CFU g^−1^ dry soil) and expressed in log_10_ units. To determine *R*. *solanacearum* densities within the aboveground plant parts, fresh stem-base samples were collected from 2 plants per treatment replicate during the year 2011 (total of 8 to 11 sampling time points depending on the crop season). Collected plants were chosen randomly at every sampling time point. Fresh segments (~1.5 to 2.0 cm) of the stem-base surface were first sterilized by dipping into 95% alcohol and flamed for 5 s. The efficiency of surface sterilization was confirmed by placing the treated parts of the stems for five minutes on a CPG plate [16] and incubating at 30°C for two days. Complete surface sterilization was achieved with this method as no visible colonies were observed on plates after two days of incubation. The segments (~1 cm) were weighed, ground and mixed into 9 mL of sterilized water. Homogenates were then diluted 10-fold and plated on M-SMSA medium. After 3 days of incubation at 30°C, bacterial densities were measured as the number of colonies per gram of stem (CFU g^−1^ fresh weight) and expressed in log_10_ units.

### Statistical analysis

Daily maximum air temperatures were obtained from Weather Online (http://www.weatheronline.co.uk/). The mean maximum temperature during each crop season was defined as *T*_*cs*_ and the mean maximum temperature during each 10-day period after transplantation as *T*_*10*_. DI data were analyzed with two-way and three-way ANOVAs with SigmaPlot 11.0 (Systat Software Inc., USA) where year, transplantation treatment and BOF treatment were used as explanatory variables. Similarly, crop season mean temperature and bacterial density data were analyzed with ANOVA as described above. Bacterial density data were log_10_-transformed before the analyses and only last sampling time point was used for analyzing bacterial densities in tomato stem base at year 2011. In all above analyses, replicate plot was included in the models as a blocking factor. Because different blocks were used between years 2011 and 2012, blocking factor × year interaction was also included in these models. Nonlinear regression analyses (Sigmoidal, Sigmoid, 3 Parameter) were used to analyze the relationships between DI and *T*_*cs*_, DI and BCE and DI and *T*_*cs*_, and to explain *R*. *solanacearum* density dynamics in tomato stems with *T*_*10*_.

## Results

### Environmental air temperature dynamics in different transplantation treatments

**Fig 1:**
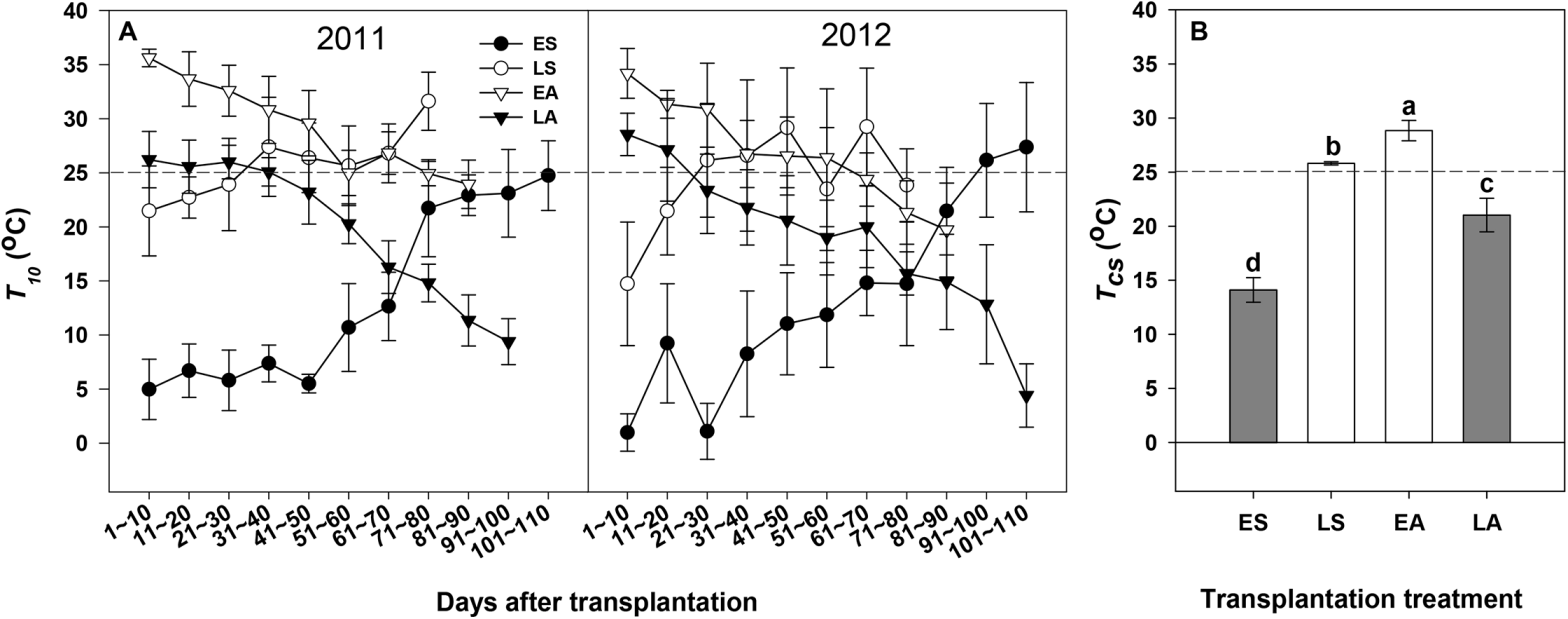
Changes in the mean maximum temperature (panel A) for every 10-day time period after transplantation (*T*_*10*_) and in the mean maximum temperature (panel B) during the entire crop season (*T*_*cs*_) for the early-spring (ES, black circle), late-spring (LS, white circle), early-autumn (EA, white triangle) and late-autumn (LA, black triangle) crop seasons transplantation treatments in 2011 and 2012. Bars show standard error of mean (SEM) in all panels. Dashed lines in all panels indicate T_*10*_ or T_*cs*_ equal to 25°C Different letters in lowercase on the top of the bar represent significance (Duncan’s multiple range test, *P* < 0.05).

The air temperature dynamics differed significantly between transplantation treatments (Fig 1). In general, *T*_*10*_ increased in ES and LS treatments over time and decreased in EA and LA treatments. The air temperatures were most of the time below 25°C in ES and LA but above 25°C in LS and EA treatments. The *T*_*cs*_ of the ES, LS, EA, and LA in 2011 and 2012 were 12.8~14.9°C, 24.9~26.0°C, 26.7~28.0°C, and 19.9~22.0°C, respectively.

### The effect of transplantation treatment on disease incidence and BOF biocontrol efficacy

**Fig 2:**
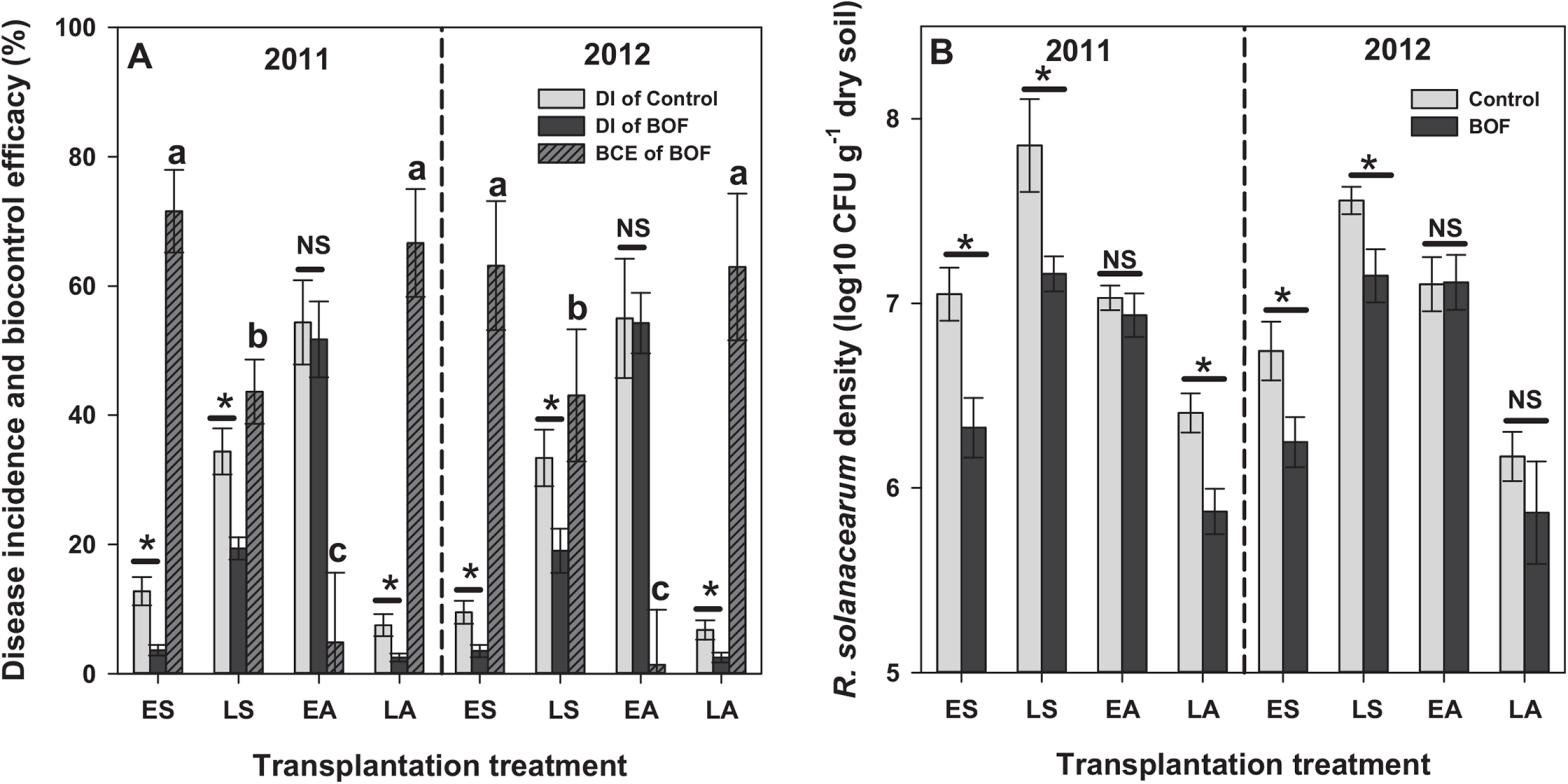
Disease incidence (DI) and biocontrol efficacy (BCE of BOF) for untreated grey bars) and BOF-treated tomato plants (bio-organic fertilizer, black bars) in the early-spring (ES), late-spring (LS), early-autumn (EA) and late-autumn (LA) transplantation treatments in 2011 and 2012. Panel B shows *R*. *solanacearum* densities in the rhizosphere soil in the beginning of harvest. In all panels, asterisks denote for statistically significant difference between BOF and control treatments (Duncan’s multiple range test, *P* < 0.05), and NS denotes for non-significant difference. Bars show SEM in all panels.

The disease incidence (DI) was significantly higher in traditional LS and EA transplantation treatments compared to experimental ES and LA transplantation treatments in the absence of BOF: DI for LS and EA crop seasons: 28.1~39.3% and 45~70%; DI for ES (6.8~16.3%) and LA (5.0~9.4%) treatments (Fig 2A, Table 1). Similarly, the biocontrol efficacy of BOF was significantly higher in experimental ES and LA transplantation treatments compared to traditional LS transplantation treatment: efficacy for ES and LA: 63.2~71.6% and 63.0~66.7% and efficacy for LS: 43.1~43.6%. BOF had no biocontrol effect in EA treatment (1.4~4.8%; non-significant; Fig 2A, Table 1). Trends of DI and the biocontrol efficacy of BOF were similar between different years (Fig 2A and Table 1).

**Table 1:** The effect of transplantation treatment, BOF treatment on bacterial wilt incidence, biocontrol efficacy of BOF and *R*. *solanacearum* densities in the rhizosphere soils and stems of tomato plants. Replicate plots were fitted as a blocking factor in the analysis.

Source	SS	df	MS	F	*P*	SS	df	MS	F	*P*
Dependent Variable	Disease incidence					Biocontrol efficacy				
Corrected Model	32430.288	23	1410.013	89.623	<0.0001	27452.725	15	1830.182	22.804	<0.0001
Intercept	42810.161	1	42810.161	2721.082	<0.0001	79769.414	1	79769.414	993.919	<0.0001
Year	1.763	1	1.763	0.112	0.739	162.885	1	162.885	2.030	0.167
Transplantation	30792.788	3	10264.263	652.413	<0.0001	26509.593	3	8836.531	110.102	<0.0001
BOF	1019.771	1	1019.771	64.818	<0.0001					
Block	58.369	4	14.592	0.928	0.455	247.227	4	61.807	0.770	0.555
Transplantation × BOF	465.991	3	155.330	9.873	<0.0001					
Year × BOF	12.700	1	12.700	0.807	0.373					
Year × Transplantation	27.749	3	9.250	0.588	0.625	79.056	3	26.352	0.328	0.805
Year × Block	46.064	4	11.516	0.732	0.574	453.965	4	113.491	1.414	0.260
Year × Transplantation × BOF	5.093	3	1.698	0.108	0.955					
Error	881.035	56	15.733			1926.179	24	80.257		
Total	761212.484	80				109148.318	40			
Corrected Total	33311.323	79				29378.904	39			
R Squared	0.974 (Adjusted value, 0.963)					0.934 (Adjusted value, 0.893)				
Dependent Variable	*Ralstonia solanacearum* densities in rhizosphere					*Ralstonia solanacearum* densities in stem				
Corrected Model	0.109	23	0.005	52.060	<0.0001	282.845	11	25.713	7.266	<0.0001
Intercept	55.147	1	55.147	603519.084	<0.0001	3001.740	1	3001.740	848.174	<0.0001
Year	0.001	1	0.001	8.562	0.005					
Transplantation	0.087	3	0.029	317.395	<0.0001	197.311	3	65.770	18.584	<0.0001
BOF	0.014	1	0.014	155.985	<0.0001	37.565	1	37.565	10.615	0.002
Block	<0.0001	4	4.244E-05	0.464	0.762	19.175	4	4.794	1.355	0.259
Transplantation × BOF	0.004	3	0.001	14.917	<0.0001					
Year × BOF	0.001	1	0.001	11.229	0.001					
Year × Transplantation	0.001	3	<0.0001	4.915	0.004					
Year × Block	0.001	4	<0.0001	1.892	0.125					
Year × Transplantation × BOF	<0.0001	3	4.576E-05	0.501	0.683	28.793	3	9.598	2.712	0.052
Error	0.005	56	9.138E-05			240.656	68	3.539		
Total	55.262	80				3525.241	80			
Corrected Total	0.115	79				523.501	79			
R Squared	0.955 (Adjusted value, 0.937)					0.540 (Adjusted value, 0.466)				

### *R*. *solanacearum* densities in tomato rhizosphere

*R*. *solanacearum* densities in the rhizosphere of BOF-treated plants were significantly lower in the ES, LS, and LA seasons in 2011 and 2012 compared to control plots (Fig 2B, Table 1). The lowest *R*. *solanacearum* densities (5.8 log10 CFU g^−1^ of dry soil) were observed in BOF-treated plants in the LA transplantation treatment (Fig 2B). A positive correlation (*P* < 0.0001) was observed between the *R*. *solanacearum* densities in rhizosphere soil and the DI, regardless of whether the analyzed plants were treated untreated with BOF (S1 Fig). Consistent with DI data, BOF effect on *R*. *solanacearum* densities was not significant in EA treatment both in 2011 and 2012, and in LA treatment in 2012 (Fig 2B, Table 1). The trends of *R*. *solanacearum* densities were similar for all four transplantation treatments. The densities were the highest in LS treatment and the lowest in LA treatment (Fig 2B).

### *R*. *solanacearum* densities in tomato plant stems

**Fig 3:**
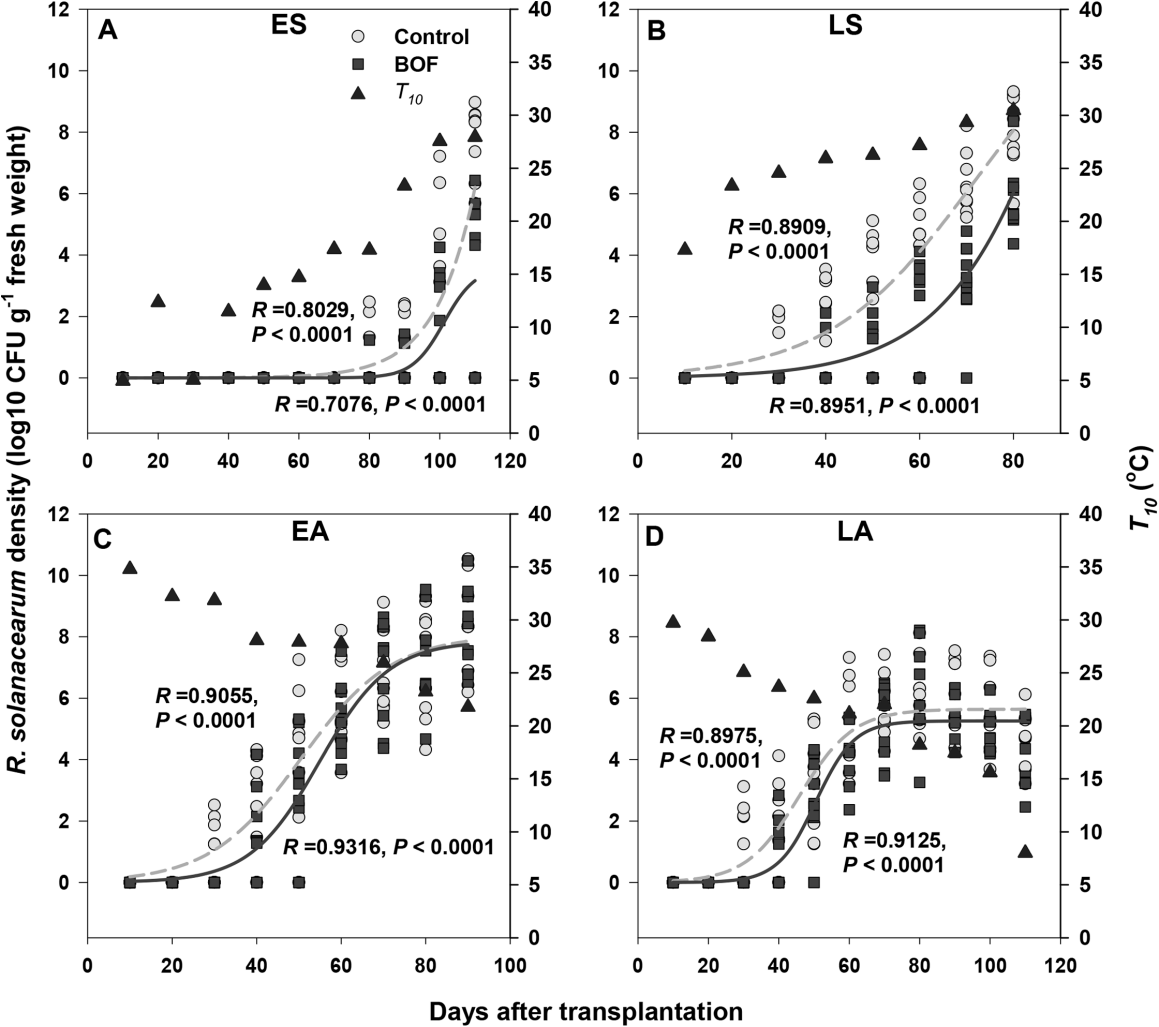
In all panels, lines denote for the dynamics of *R*. *solanacearum* densities in the stem-bases of tomato plants in control (grey lines for means and grey circles for replicates) and BOF (black lines for means and black squares for replicates) treatments in the early-spring (panel A), late-spring (panel B), early-autumn (panel C) and late-autumn (panel D) transplantation treatments in year 2011. The increase in *R*. *solanacearum* densities over time was fitted with nonlinear regression analysis (Equation: Sigmoidal, Sigmoid, 3 Parameter). The triangles show the average maximum air temperatures for each 10-day period after transplantation.

The *R*. *solanacearum* densities in the tomato stems were affected by both transplantation and BOF treatments (Fig 3, Table 1). In the ES treatment (Fig 3A), *T*_*10*_ stayed below 18°C until 70 days after transplantation, and no *R*. *solanacearum* was detected in either BOF-treated or untreated plants. Once *T*_*10*_ exceeded 20°C, *R*. *solanacearum* densities increased sharply. Pathogen densities were reduced approximately 50% in BOF-treated plants: 6.21 log10 CFU g^−1^ versus 3.16 log10 CFU g^−1^ bacteria per fresh weight of stem for control and BOF-treated plants, respectively. In the LS treatment (Fig 3B), *T*_*10*_ increased from 20 to 31°C 20 d after transplantation and *R*. *solanacearum* densities increased quickly during this period. Finally, we observed 8.04 log10 CFU g^−1^ and 6.08 log10 CFU g^−1^ bacteria per fresh weight of stem for control and BOF-treated plants, respectively. In the EA treatment (Fig 3C), *T*_*10*_ was above 25°C throughout the first 70 days after transplantation, and *R*. *solanacearum* densities increased sharply during this period. During the final 20 days, *T*_*10*_ remained below 25°C and *R*. *solanacearum* densities increased only slightly. Pathogen densities reached approximately 8.0 log10 CFU g^−1^ bacteria per fresh weight of stem in both control and BOF-treatments. In the LA treatment (Fig 3D), *T*_*10*_ was above 20°C throughout the first 70 days after transplantation and decreased sharply below 10°C for the final 20 days. The *R*. *solanacearum* density dynamics were similar to those in the EA treatment, with the exception that *R*. *solanacearum* densities were generally lower.

### Correlation between disease incidence and temperature variation in different experimental treatments

**Fig 4:**
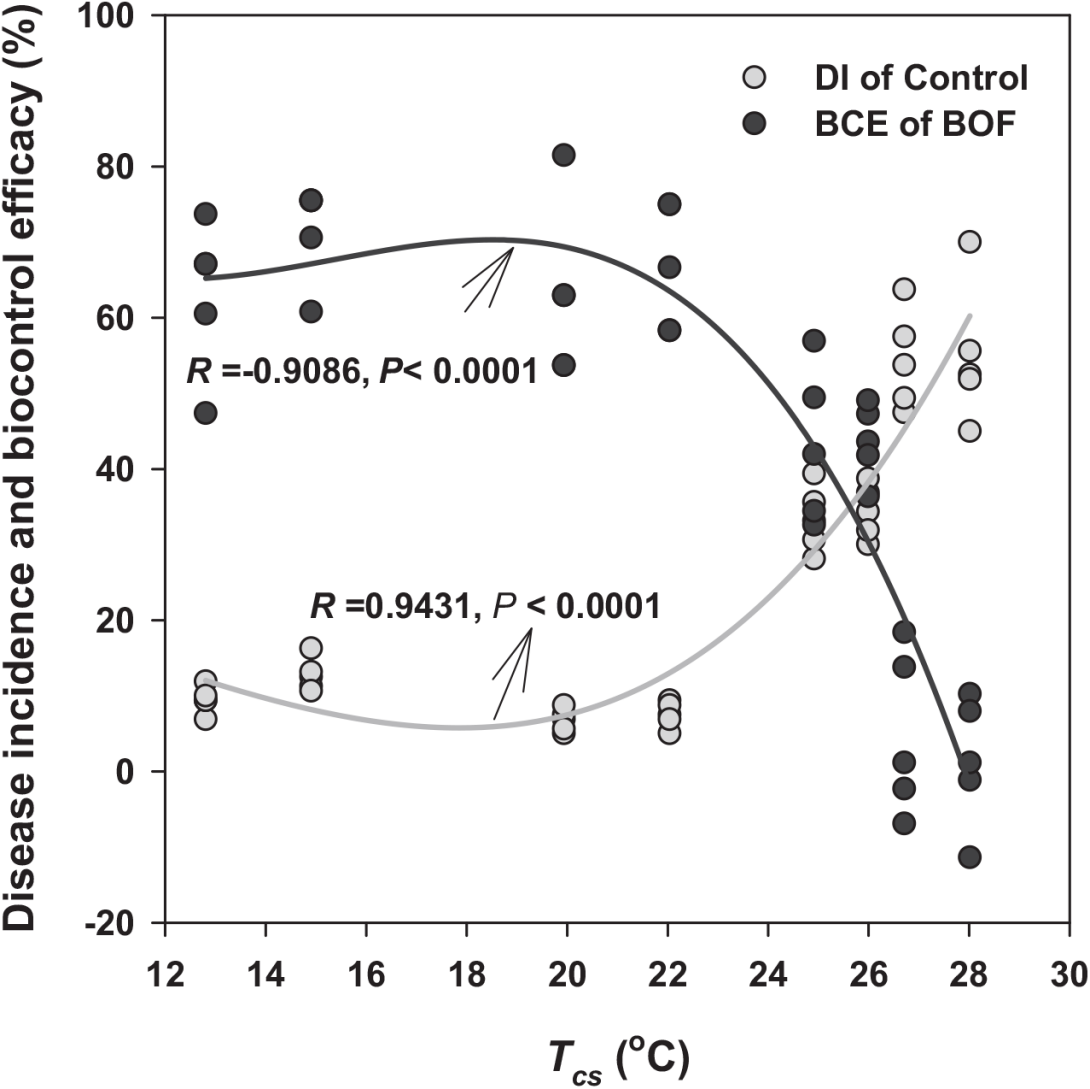
Nonlinear regression analyses (Equation: Sigmoidal, Sigmoid, 3-Parameter) between mean maximum temperature averaged over crop season (*T*_*cs*_) and both biocontrol efficacy of BOF (black line) and disease incidence in control treatment (grey line).

The *T*_*cs*_ correlated significantly with both DI (*R* = 0.9431, *P* < 0.0001, positive correlation) and BCE (*R* = -0.9086, *P* < 0.0001; negative correlation Fig 4). When *T*_*cs*_ ranged from 12°C to 23°C, the DIs in both control and BOF-treated plants were approximately 10%, while the BOF biocontrol efficacy ranged from 60% to 70% (Fig 4). As *T*_*cs*_ increased to 26°C, the DI increased to approximately 35%, and the BCE decreased to 45% (Fig 4). When *T*_*cs*_ exceeded 28°C, the DI increased to 55%. Under such conditions, BOF lost its biocontrol efficacy (Fig 4).

### The effect of transplantation and BOF treatments on tomato plant yield and income

Transplanting time and BOF application both had significant effects on the tomato yield and farmer income (Tables 2 and 3). While preponing transplantation time from LS to ES decreased tomato yield, postponing transplantation time from EA to LA increased tomato yield (Tables 2 and 3). Despite the reduced yield of ES treatment, farmer earnings were compensated by higher tomato prices leading to higher total income (Table 2). Applying BOF generally increased both the yield and income except for the EA transplantation treatment (Table 2).

**Table 2:** The effect of transplantation time and BOF treatment on tomato yield and farmer’s income (means ± SEM). Yield denotes for mean yield for five replicate plots per treatment. Price and income are shown in Chinese Yuans (RMB). The price of tomato denotes for market price of the time based on farmer’s accounting.

		Price (RMB kg^−1^)	Actual Yield (ton ha^−1^)		Income (10^3^ RMB ha^−1^)	
			Control	BOF	Control	BOF
2011	ES	5.0	38.39±0.96	42.41±0.36	191.95±4.82	212.03±1.79
	LS	3.0	49.88±2.71	61.28±1.30	149.63±8.12	183.83±3.90
	EA	4.0	38.33±5.48	40.53±4.93	153.30±21.92	162.12±19.7
	LA	4.0	55.50±1.03	58.50±0.38	222.00±4.11	234.00±1.5
2012	ES	5.5	43.44±0.86	46.32±0.45	238.92±4.72	254.76±2.50
	LS	3.5	53.30±3.49	64.80±2.73	186.55±12.22	226.80±9.55
	EA	4.5	39.60±8.13	40.26±4.12	178.20±36.58	181.17±18.55
	LA	4.5	59.68±0.95	62.40±0.49	268.56±4.30	280.80±2.20

**Table 3:** The effect of transplantation time, BOF treatments and Blocks on tomato yield and income.

Dependent Variable	Actual Yield					Income				
Source	SS	df	MS	F	P	SS	df	MS	F	P
Corrected Model	0.549	23	0.024	17.558	<0.0001	0.566	23	0.025	18.079	<0.0001
Intercept	1757.647	1	1757.647	1292360.971	<0.0001	2254.544	1	2254.544	1656887.829	<0.0001
Year	0.014	1	0.014	10.278	0.002	0.125	1	0.125	91.993	<0.0001
Transplantation	0.473	3	0.158	115.966	<0.0001	0.379	3	0.126	92.790	<0.0001
BOF	0.034	1	0.034	24.723	<0.0001	0.034	1	0.034	24.693	<0.0001
Block	0.005	4	0.001	0.932	0.452	0.005	4	0.001	0.933	0.452
Transplantation × BOF	0.015	3	0.005	3.736	0.016	0.015	3	0.005	3.735	0.016
Year × BOF	<0.0001	1	<0.0001	0.284	0.596	<0.0001	1	<0.0001	0.284	0.596
Year × Transplantation	0.005	3	0.002	1.122	0.348	0.004	3	0.001	1.062	0.373
Year × Block	0.003	4	0.001	0.566	0.688	0.003	4	0.001	0.565	0.689
Year × Transplantation × BOF	<0.0001	3	4.09E-05	0.030	0.993	<0.0001	3	4.084E-05	0.030	0.993
Error	0.076	56	0.001			0.076	56	0.001		
Total	1758.272	80				2255.186	80			
Corrected Total	0.625	79				0.642	79			
R Squared	0.878 (Adjusted value, 0.828)					0.881 (Adjusted value, 0.833)				

## Discussion

Here we studied experimentally if we can reduce bacterial wilt disease incidence and increase the BOF performance by simply altering the traditional seedling transplantation times of tomato. We found that both preponing and postponing traditional transplantation times to the cooler period significantly decreased bacterial wilt disease incidence, increased BOF biocontrol efficacy especially during the late-autumn crop season, and led to lower *R*. *solanacearum* pathogen densities in both tomato plant rhizosphere and tomato base stems. Correlation analyses indicated that these effects could be attributed to lower environmental temperatures during the experimental ES and LA crop seasons. Even though preponing transplantation time of spring crop season reduced the yield of tomato, this was financially compensated by relatively higher market prices leading to increased farmer income. Postponing transplantation time of autumn season increased both yield and farmer income. Altering traditional tomato transplantation times could thus offer simple but effective way to reduce bacterial wilt incidence and improve the effectiveness of BOF in the field setting.

Why did bacterial wilt incidence decrease with the lower environmental temperature? Several reports have shown that low temperatures can directly affect the virulence of *R*. *solanacearum* reducing or preventing the root colonization and invasion potentially via attenuated or lost twitching motility [26]. It has been also found that the expression of global virulence regulators, *HrpG* and *HrpB*, is temperature-regulated, and depending on the pathogen strain, can be positively correlated with increasing temperature [27,28]. Consistent with this, we observed a significantly positive correlation between mean crop season temperature and disease incidence. This suggests that high environmental temperature might be essential for triggering *R*. *solanacearum* virulence in the field. Temperature fluctuations are also known to affect *R*. *solanacearum* survival and persistence in the soil between different crop seasons. For example, Scherf et al. [28] reported that temperature fluctuations played a critical role for *R*. *solanacearum* R3B2 and native U.S. strain viability as both strains were able to survive at -20°C in infected geranium tissue for at least 6 months as long as the temperature was kept constant. In the present study, we observed relatively wider temperature fluctuations in experimental ES and LA transplantation treatments. As a result, occasional temperature drops below 18°C could have reduced pathogen viability, which could partly explain reduced disease incidence via lowered *R*. *solanacearum* densities in the soil.

Altering traditional tomato transplantation time also improved the efficacy of BOF biocontrol especially during the late-autumn crop season. *B*. *amyloliquefaciens* is able to produce broad-spectrum antibiotics that often protect plants either by directly antagonizing pathogens or indirectly by inducing systemic resistance in plants [29]. Establishment of such antagonists may then promote the root-colonization capacity of the antagonist and prevent the colonization of invading plant pathogens [30–32]. It has previously been shown that *B*. *amyloliquefaciens* exerts direct antagonism towards *R*. *solanacearum* in the laboratory conditions [13,33,34]. This could explain why BOF treated plants harbored generally lower *R*. *solanacearum* densities both in the soil rhizosphere and aboveground tomato stem base. However, BOF had no biocontrol efficacy during EA transplantation treatment. First, it is possible that elevated temperature directly reduced the biocontrol efficacy of *Bacillus*-fortified BOF. Earlier studies have shown that the production of antibiotic molecules by *Bacillus* bacteria depends on the environmental temperature [16]. Alternatively, elevated temperature could have affected *R*. *solanacearum* resistance to these antibiotics [23,24]. Even though pathogen densities in the EA rhizosphere were similar to other transplantation treatments, pathogen densities within the plant stems were the highest in the EA treatment. As a result, the BOF biocontrol efficacy could have depended on *R*. *solanacearum* density, resulting in more frequent pathogen invasion to xylem in EA transplantation treatment. While these hypotheses cannot be answered with the current data, additional experiments are on their way to study the role of elevated temperature for BOF biocontrol efficacy.

In addition to reducing bacterial wilt disease incidence, alternating traditional tomato transplantation times had positive effects on farmer total income. Even though preponing the tomato transplantation time in the spring season reduced the tomato yield, relatively higher tomato market prices were able to compensate the farmer income for the ES transplantation treatment. Postponing the autumn crop clearly increased tomato yield by reducing crop losses to bacterial wilt and by increasing BOF biocontrol efficacy. As a result, later transplantation time clearly increased farmer income during the second crop season. The late autumn crop was also preferred over the early spring crop, because early transplantation time overlapped with Chinese New Year. Even though some between-year variation was observed, the significant effect of transplanting time was similar over the two-year trial period. This has convinced the farmer who participated in this study to exclusively apply LA transplantation time for the second tomato crop.

In conclusion, our results demonstrate that simple alteration of tomato transplantation time to cooler periods can considerably lower bacterial wilt disease incidence. In addition to having positive immediate effects on tomato yield and the farmer’s income, reducing pathogen densities in the soil is likely to have positive long-term effects via reduced disease outbreak risk in the future. Postponing transplantation time also increased the BOF biocontrol efficacy, which suggests that biocontrol agents might be also affected by environmental temperature. We hope to resolve more detailed mechanisms behind temperature and pathogen-host and pathogen-biocontrol agent interactions in the future.

## Supporting Information

S1 Fig Linear regression analysis between disease incidence (log_10_-transformed) and the *R*. *solanacearum* densities in the tomato rhizosphere soils for untreated (white circles) or BOF-treated (black circles) plants on the first date of harvest (graph includes all transplantation treatments on years 2011 and 2012). (DOCX)

S1 Table Transplantation and harvest dates for different transplantation treatments in 2011 and 2012. (DOCX)
