# Supplementary material for: Altering Transplantation Time to Avoid Periods of High Temperature Can Efficiently Reduce Bacterial Wilt Disease Incidence with Tomato
Source: PLoS One. 2015 Oct 6;10(10):e0139313. doi: 10.1371/journal.pone.0139313 (PMC4595502; doi:10.1371/journal.pone.0139313)
Supplement: S1 Fig — (DOCX) [file pone.0139313.s001.docx]

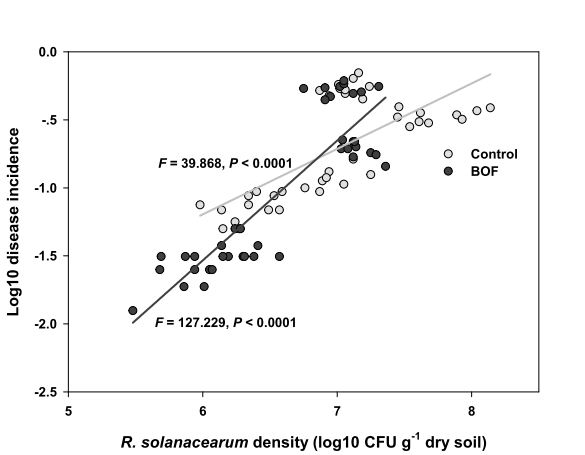


S1 Fig. Linear regression analysis between disease incidence (log_10_-transformed) and the *R. solanacearum* densities in the tomato rhizosphere soils for untreated (white circles) or BOF-treated (black circles) plants on the first date of harvest (graph includes all transplantation treatments on years 2011 and 2012).
