# Supplementary material for: Altering Transplantation Time to Avoid Periods of High Temperature Can Efficiently Reduce Bacterial Wilt Disease Incidence with Tomato
Source: PLoS One. 2015 Oct 6;10(10):e0139313. doi: 10.1371/journal.pone.0139313 (PMC4595502; doi:10.1371/journal.pone.0139313)
Supplement: S1 Table — (DOCX) [file pone.0139313.s002.docx]

Supplementary Table 1. Transplantation and harvest dates for different transplantation treatments in 2011 and 2012.

|  | 2011 | | 2012 | |
| --- | --- | --- | --- | --- |
| Transplantation  Treatment | Transplantation  date | First harvest  date | Transplantation  date | First harvest  date |
| Early-spring (ES) | 22/01 | 02/05 | 15/01 | 03/05 |
| Late-spring (LS) | 02/04 | 20/06 | 29/03 | 15/06 |
| Early-autumn (EA) | 23/07 | 15/10 | 20/07 | 13/10 |
| Late-autumn (LA) | 28/08 | 7/12 | 03/09 | 10/12 |
